# Hepatoprotective effect of silyamarin in individuals chronically exposed to hydrogen sulfide; modulating influence of TNF-α cytokine genetic polymorphism

**DOI:** 10.1186/2008-2231-21-28

**Published:** 2013-04-08

**Authors:** Ali Mandegary, Arastoo Saeedi, Aziz Eftekhari, Vahideh Montazeri, Elham Sharif

**Affiliations:** 1 Department of Pharmacology & Toxicology, School of Pharmacy and Pharmaceutics Research Center, Kerman University of Medical Sciences, Kerman, Iran; 2 Physiology Research Center, Kerman University of Medical Sciences, Kerman, Iran; 3 Herbal and Traditional Medicines Research Center, Kerman University of Medical Sciences, Kerman, Iran

**Keywords:** Silymarin, Hepatoprotective, Hydrogen sulfide, Occupational toxicology, Polymorphism

## Abstract

**Background and the purpose of the study:**

Silymarin, a standardized extract of the milk thistle (*Silybum marianum*), is believed to exert some of its hepatoprotective effects though inhibition of free radicals and inflammation. In this study the effect of some pro- and anti-inflammatory cytokines and also antioxidant genes polymorphisms on the hepatoprotective effects of silymarin in the occupationally exposed individuals to hydrogen sulfide (H_2_S) in the sour natural gas refinery was investigated.

**Methods:**

We genotyped seven polymorphisms in six genes reported by others as modifiers of oxidative stress (NQO1, mEPXH1, GSTT1 and GSTM1) and inflammation (TNF-α and TGF-β1) for an association in effect of decreasing in liver function tests (LFTs). The LFTs of 77 sour gas refinery workers were measured before and after administration of silymarin (140 mg, three times per day for 1 month).

**Results:**

A significant reduction of blood AST, ALT and ALP was observed after 30 days of consumption (p < 0.001). The decreasing effect of silymarin on ALT in the subjects with high producer genotype (A allele carriers) was less than low producers. There were no significant associations between TGF-β1 and the studied genes of oxidative stress pathway and the effectiveness of silymarin.

**Conclusion:**

This is the first report about the effectiveness of silymarin in the subjects exposed chronically to H_2_S. Meanwhile, the modulatory effect of TNF-α on the effectiveness of silymarin might be used for individualize therapy.

## Introduction

The workers of natural gas refineries are exposed to potentially toxic substances like hydrogen sulfide (H_2_S) and benzene. Recently we have shown that the oxidative stress biomarkers increased in the workers of sweet and, in higher levels, sour gas refineries [1]. Meanwhile the levels of liver function tests (LFTs) were high in the H_2_S exposed subjects [1]. Although pathogenesis of liver toxicity induced by H_2_S has not been elucidated so far, there is experimental evidence that induction of oxidative stress plays a key role [2,3]. Several observations suggest that increased reactive oxygen species (ROS) and resulting oxidative stress is a critically important determinant of liver injury [4,5]. Oxidative stress is involved in the release of pro-inflammatory mediators (cytokines and chemokines) and injury to the liver cells [4,5]. Silymarin, a mixture of flavonolignans isolated from *Silybum marianum* (milk thistle), has been used to treat liver diseases for hundreds of years [6,7]. The hepatoprotective effect of silymarin is mainly due to its strong antioxidant activity and scavenging the free radicals [8]. It also effectively inhibits the inflammation [9,10], which can also damage the liver [6,11]. Silybin is an effective antioxidant, conserving GSH in liver cells while stabilizing the liver cell membranes against oxidative attack [4,12].

There are inter-individual variations in detoxifying enzymes and cytokines that could be translated into differences in susceptibility for xenobiotic toxicity and treatment outcomes [13-17]. So in this study, we decided to investigate the association of polymorphisms in the enzymes involved in general oxidative stress defence including NAD(P)H:quinone oxidoreductase-1 (NQO1), Glutathione S-transferases (GST) M1 and T1, and microsomal epoxide hydrolase (EPHX1) enzymes and the two most important cytokines involved in the pathogenesis of liver disease with the basis of inflammation, namely tumor necrosis factor-α (TNF-α) and transforming growth factor-β1 (TGF-β1) cytokines and the treatment outcome of silymarin in the subjects chronically exposed to H_2_S.

## Materials and methods

### Materials

Silymarin containing tablets (Livergol) was purchased from Goldaru, Isfahan, Iran. PCR master mix was obtained from CinnaClone (Iran). Ethylenediamine tetraacetic acid (EDTA), Tris, agarose, Sodium Dodecyl Sulfate (SDS), isopropanol and ethanol were purchased from Merck (Germany). Proteinase K and DNA ladder were obtained from Fermentas (Germany).

### Study subjects

In this cross-sectional study, 77 employees of sour gas refinery were recruited. According to local authorities, the atmospheric concentration of H_2_S in the place was 10–15 ppm [1]. The eligibility criteria for the cases were as follow: working at least 5 years in the refinery and no history of liver disease. Demographic data, general health conditions, lifestyle, years of employment and smoking habits were registered in a questionnaire. Written informed consent was obtained from the subjects after describing the aim of the study. The study was conducted according to the declaration of Helsinki and subsequent revisions and approved by the Ethical Committee of Kerman University of Medical Sciences (No: 91/91/k, KMU). The liver function tests (LFTs) including aspartate aminotransferase (AST) and alanine aminotransferase (ALT) and alkaline phosphatase (ALP) were determined before and after administration of silymarin at the dosage of 150 mg three times per day (Livergol, Goldaru, Esfahan, Iran).

### DNA extraction and GST genotyping

DNA was extracted from 5 ml peripheral blood using Miller method [18]. The subjects were genotyped for the deletion in GSTM1 and GSTT1 by a multiplex polymerase chain reaction (PCR). The PCR conditions and primers were as described previously [19]. TNF-α -308G/A and TGF-β1 codon 10 and codon 25 genotyping methods were based on the PCR-ARMS, and were similar to those previously described [20]. The amplified fragments were 184-bp, 241-bp and 233-bp respectively for TNF-α, TGF-β1 codon 10 and 25. EPXH1 exon 4 (His139Arg), NQO1 (C609T) and TNF-α (G-308A, for confirmation of PCR-ARMS for TNF-α -308G/A) genotyping methods were based on PCR-RFLP and as described before [21-23]. The products of each PCR reaction and digestion were resolved by electrophoresis on 3% agarose gels stained with ethidium bromide. Reliability and validity of the PCR and RFLP methods were assessed through re-conducting the genotype assays using at least a 10% sample of our DNA samples. The polymorphisms of heterozygous, homozygous and wild-type TNF-α which obtained by PCR-ARMS were re-checked with PCR-RFLP method. In addition, for TGF-β1 the method was also assessed through re-conducting the genotype assays using most of DNA samples with mutations (both heterozygous and homozygous). The results for all re-assessments were 100% concordant.

### Statistical analysis

For the comparison of continuous variables, we firstly checked the assumption that they were normally distributed. If the distribution was normal, the results were expressed as means and standard error (SE) and a classical t-test or one-way ANOVA was used accordingly. According to the genes polymorphisms, genotypes were collapsed into two categories: homozygous (common genotypes) versus heterozygous + variant homozygous and they were coded as follow: GSTM1*0 (code 0) and GSTM1-positive (code 1), GSTT1*0 (code 0) and GSTT1-positive (code 1), NQO1 CC allele (code 0) and CT/TT alleles (code 1), EPXH exon 4 AA allele (code 0) and AG/GG alleles (code 1), TGF-β1 codon 10 TT allele (code 0, high producer) and TC/CC alleles (code 1, low producer), and TGF-β1 codon 25 GG allele (code 0, high producer) and GC/CC alleles (code 1, low producer). Combination of TGF-β1 codons 10 and 25 was as follow: TT/GG (code 0, high producer) and all other combinations (code 1, low producer). Simple and multiple linear regression was used to screen the independent variables including genetic polymorphism to best predict the value of the LFTs and LFTs difference. Age and BMI variables believed to potentially confound the association with LFTs’ level were included in the multivariate model. The significance (P) of genetic polymorphisms factors from multivariate analysis was considered as adjusted p-value. Differences between LFTs before and after administration of silymarin were analyzed using paired-sample t-test. For all the tests, a p-value less than 0.05 was considered to be significant. All analyses were conducted using SPSS statistical software (version 11.5).

## Results

**Figure 1:**
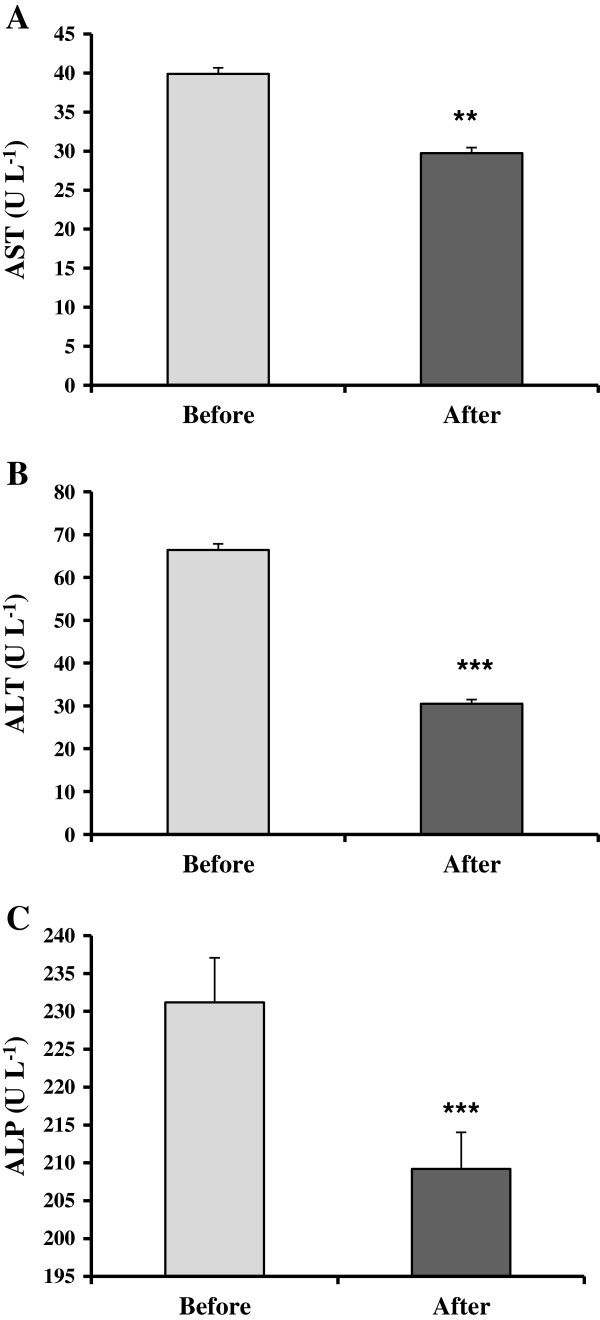
**Effect of the consumption of silymarin on serum LFTs.** AST (**A**), ALT (**B**) and ALP (**C**) levels in the chronically H_2_S exposed subjects (n = 77) were measured before and after administration of silymarin. Results are expressed as mean ± SE. ** *p* < 0.001 and *** *p* < 0.001 compared with before treatment by Pair sample t-test.

Significant decreases in AST (P < 0.01), ALT (P < 0.001) and ALP (P < 0.001) were observed by use of silymarin (Figure 1). The mean ± SE of AST before and after using were 39.8 ± 0.78 and 29.7 ± 0.07 U L^-1^ (Figure 1A). A significant (P < 0.001) decrease in ALT was observed after administration of silymarin (66.4 ± 1.4 U L^-1^ before versus 30.5 ± 0.9 U L^-1^ after (Figure 1B). After use of silymarin, the ALP level decreased significantly (P < 0.001). The mean ± SE values before and after were 231.2 ± 5.9 and 209.2 ± 4.8 U L^-1^ (Figure 1C).

Simple and multivariate linear regression were performed to analyze the association of GSTM1, GSTT1, NQO1 and EPXH1 genes polymorphisms with changes in LFTs after administration of silymarin (Table 1). There was no association between the abovementioned genes’ polymorphisms and decreasing in LFTs after administration of silymarin.

**Table 1:** Association of oxidative stress related genes polymorphisms with changes in LFTs’ levels by consumption of silymarin

**LFT**	**Gene**	**Unadjusted**		**Adjusted**	
		**B (CI)**	**p-value**	**B (CI)**	**p-value**
**AST**	**NQO1**	−0.73 (−3.38, 4.84)	0.72	−0.05 (−4.10, 3.92)	0.98
	**GSTM1**	−0.58 (−4. 02, 3.03)	0.75	−0.64 (−4.25, 2.97)	0.72
	**GSTT1**	1.27 (−3.10, 5.64)	0.56	1.92 (−2.55, 6.38)	0.40
	**EPXH4**	1.34 (−3.10, 5.77)	0.54	1.72 (−2.73, 6.17)	0.44
**ALT**	**NQO1**	0.87 (−4.20, 5.92)	0.73	0.79 (−4.40, 5.98)	0.76
	**GSTM1**	−4.24 (−8. 62, 0.13)	0.05	4.21 (−8.67, 0.25)	0.06
	**GSTT1**	1.30 (−4.23, 6.83)	0.64	1.22 (−4.50, 6.92)	0.65
	**EPXH4**	1.04 (−3.02, 4.67)	0.54	0.89 (−4.46, 6.24)	0.74
**ALP**	**NQO1**	−12.83 (−39.89, 14.22)	0.35	−2.64 (−20.22, 14.95)	0.76
	**GSTM1**	1.21 (−23.79, 26.21)	0.92	2.27 (−13.67, 18.22)	0.77
	**GSTT1**	21.28 (−8.93, 51.50)	0.16	10.21 (−9.46, 29.87)	0.30
	**EPXH4**	−14.20 (−47.62, 19.22)	0.40	1.73 (−17.78, 21.24)	0.86

Multivariate regression: Parameters adjusted for age and BMI;

B: Partial regression coefficient.

Simple and multivariate linear regression were performed to analyze the association of TNF-α, TGF-β1 codon 10, TGF-β1 codon 25 and combination of these two codons polymorphisms with changes in LFTs after administration of silymarin (Table 2). TNF-α G-308A polymorphism was significantly associated with deceasing in the AST (p = 0.04) and ALT (p = 0.003). These results show that decreasing in the AST and ALT levels are lower in the subjects with high producer TNF-α genotype. These associations remained significant after adjustment with age and BMI. There was also negative significant association between TGF-β1 codon 10 polymorphisms and decreasing in ALP that this association was abolished when age and BMI added to the model.

**Table 2:** Association of inflammation related cytokines polymorphisms with changes in LFTs’ levels by consumption of silymarin

***LFT***	***Gene***	***Unadjusted***		***Adjusted***	
		**B (CI)**	**p-value**	**B (CI)**	**p-value**
**AST**	TNF-α	−4.27 (−8.57, 0.03)	0.05	−4.58 (−8.97, -0.21)	0.04*
	TGF-β1 codon 10	−0.77 (−5. 17, 3.61)	0.72	−0.07 (−4.56, 4.41)	0.97
	TGF-β1 codon 25	2.78 (−3.36, 8.93)	0.36	2.79 (−3.40, 8.98)	0.37
	TGF-β1 codons 10/25	0.99 (−3.04, 5.03)	0.62	1.37 (−2.73, 5.46)	0.50
**ALT**	TNF-α	−8.30 (−13.73, -2.86)	0.003*	−8.93 (−14.62, -3.23)	0.003*
	TGF-β1 codon 10	−3.62 (−8.92, 1.67)	0.17	−3.43 (−9.00, 2.14)	0.22
	TGF-β1 codon 25	5.87 (−1.29, 13.03)	0.10	5.94 (−1.36, 13.24)	0.10
	TGF-β1 codons 10/25	−0.33 (−5.28, 4.63)	0.89	0.03 (−5.11, 5.24)	0.99
**ALP**	TNF-α	−6.17 (−41.08, 28.75)	0.72	−0.85 (−21.93, 20.21)	0.93
	TGF-β1 codon 10	−31.45 (−61.31, -1.59)	0.03	−9.05 (−27.43, 8.32)	0.33
	TGF-β1 codon 25	16.67 (−25.38, 58.73)	0.43	−12.00 (−37.17, 13.17)	0.34
	TGF-β1 codons 10/25	−16.16 (−44.41, 12.07)	0.25	−8.65 (−24.63, 7.33)	0.28

Multivariate regression: Parameters adjusted for age and BMI. *Significant effect. B: Partial regression coefficient.

## Discussion

In light of our recent study demonstrating increase in the LFT levels in the workers exposed chronically to H_2_S, we became interested in determining whether a well-known hepatoprotective natural product like silymarin could decrease the LFTs in the subjects and if the genetics of the subjects could determine responding to treatment. Administration of silymarin (140 mg tablets, thrice per day for 1 month) significantly reduced the LFTs in the subjects. The hepatoprotective mechanisms of silymarin on H_2_S-induced elevation of LFTs have not been addressed so far. Recently we have also shown that stress oxidative biomarkers were higher in the workers in exposure to H_2_S [1]. Lines of evidence demonstrate that stress oxidative is one of the main mechanisms for the toxic effect of xenobiotics on the liver [4,15,24]. Antioxidant activity and scavenging the free radicals have been reported as the main mechanism for the hepatoprotective effect of silymarin [6].

Our data showed significant association (B = −8.30, CI: -13.73,-2.86) between the polymorphism of TNF-α G-308A and the changes in ALT and AST after administration of silymarin (Table 2). In other words, decrease in ALT and AST in the high TNF-α producers treated with silymarin is significantly lower than low producers. TNF-α is a pro-inflammatory cytokine involved in the pathogenesis of variety of liver injuries [25]. TNF-α, in concert with other cytokines, mediates the recruitment of neutrophils to the liver that induce inflammation and cell death [26]. Production of TNF-α is under genetic control which determined by a number of single nucleotide polymorphisms in this gene. The G-308A polymorphism in the promoter of the gene is the most studied one that has been linked to higher TNF-α production [27,28] as homozygosity for TNF1 (TNF1/TNF1) is associated with low production (TNF-α^Lo^), whereas the TNF1/TNF2 and TNF2/TNF2 genotypes are high producers (TNF-α^Hi^). The effect of TNF-α on the production of ROS was also shown elsewhere [25]. TNF-induced ROS production activates JNK (c-jun N-terminal kinase) by oxidizing and inactivating several members of the MAP kinase phosphates (MKPs). Taking together, one might relate the less hepatoprotective effect of an antioxidant compound like silymarin in high producer of TNF-α to higher production of ROS in these subjects.

Our data also showed no association between the polymorphisms of TGF-β and some of detoxifying enzymes and the changes in LFTs after administration of silymarin. Univariate regression analysis showed a significant association between TGF-β1 gene polymorphism at codon 10 and ALP levels that this association was abolished after adjusting the regression with confounders like age and BMI. This is in consistent with the previous reports about the role of BMI on the LFTs [29].

## Conclusion

Administration of silymarin (140 mg tablets, thrice per day for 1 month) significantly reduced the LFTs in the subjects exposed chronically to H_2_S. Meanwhile, increasing the dose of silymarin in the high TNF-α producers to obtain therapeutic effects might be needed.

## Competing interests

The authors declare that they have no competing interests.

## Authors’ contributions

AM participated in the design of the study, performed the statistical analysis, participated in the sequence alignment and drafted the manuscript. AS carried out sampling and carried out genotyping of some genes. AE carried out genotyping of some genes and filed the data. VM carried out genotyping of some genes. ESh carried out DNA extraction and helped to draft the manuscript. All authors read and approved the final manuscript.
